# Image quality to estimate ventricular ejection fraction by last year medical students improves after short courses of training

**DOI:** 10.1186/s12909-019-1809-2

**Published:** 2019-10-22

**Authors:** Tobias Hüppe, Heinrich Volker Groesdonk, Thomas Volk, Stefan Wagenpfeil, Benedict Wallrich

**Affiliations:** 1 Department of Anesthesiology, Intensive Care and Pain Therapy, Saarland University, Medical Center, Kirrberger Straße 100, 66421 Homburg, Saar Germany; 2 Department of Intensive Care Medicine, Helios Klinikum Erfurt, Erfurt, Germany; 3 Institute for Medical Biometry, Epidemiology and Medical Informatics, Saarland University Medical Center, Homburg, Saar Germany

**Keywords:** Echocardiography, Training program, Students, Left ventricular ejection fraction, Ultrasound

## Abstract

**Background:**

Transthoracic echocardiography is the primary imaging modality for diagnosing cardiac conditions but medical education in this field is limited. We tested the hypothesis that a structured theoretical and supervised practical course of training in focused echocardiography in last year medical students results in a more accurate assessment and more precise calculation of left ventricular ejection fraction after ten patient examinations.

**Methods:**

After a theoretical introduction course 25 last year medical students performed ten transthoracic echocardiographic examination blocks in postsurgical patients. Left ventricular function was evaluated both with an *eye-balling method* and with the *calculated ejection fraction* using diameter and area of left ventricles. Each examination block was controlled by a certified and blinded tutor. Bias and precision of measurements were assessed with Bland and Altman method.

**Results:**

Using the *eye-balling method* students agreed with the tutor’s findings both at the beginning (88%) but more at the end of the course (95.7%). The variation between student and tutor for *calculation* of area, diameter and ejection fraction, respectively, was significantly lower in examination block 10 than in examination block 1 (each *p* < 0.001). Students underestimated both the length and the area of the left ventricle at the outset, as complete imaging of the left heart in the ultrasound sector was initially unsuccessful.

**Conclusions:**

A structured theoretical and practical transthoracic echocardiography course of training for last year medical students provides a clear and measurable learning experience in assessing and measuring left ventricular function. At least 14 examination blocks are necessary to achieve 90% agreement of correct determination of the ejection fraction.

## Background

Transthoracic echocardiography is the primary imaging modality for diagnosing cardiac conditions. Although cardiac ultrasound has become standard imaging in many disciplines today, the standards and goals to teach remain still undefined. The inclusion of cardiac ultrasound training in undergraduate medical training may be advantageous for many disciplines because cardiac risk stratification is becoming more important in increasingly aging patients.

Cardiac ultrasound has been used successfully to teach cardiac anatomy and physiology to medical students [1–4]. Moreover, structured echocardiographic education in students provides measurable increasing skills in image acquisition [5–7], volume assessment [8], cardiomyopathies [9–13], valve dysfunction [11, 13–15], and pericardial effusion [12, 13]. Furthermore, although inferior to teaching by an expert cardiographer, hands-on training by student tutors led to a significant gain in echocardiography skills in 4th and 5th year students [16]. Even the use of simulations as a supplement to traditional educational approaches improves learning among medical students [17]. However, it is still unclear whether such trainings improve the correct determination of the ejection fraction as, in addition to the velocity time integral (VTI) [18] or the global longitudinal strain (GLS) [19], one of the most commonly used surrogate parameters of the left ventricular systolic pump function [19].

We, therefore, specifically tested the hypothesis that a structured theoretical and supervised practical course of training in focused echocardiography for last year medical students leads to significant more accurate assessments and more precise calculations of the left ventricular ejection fraction.

## Methods

### Student selection

We enrolled 25 last year medical students during their one-year internship without any previous theoretical and practical experience in transthoracic echocardiography into the study. After consultation with the local ethics committee, an ethics approval was not necessary.

### Protocol

All students received a 3 h introduction course in focused transthoracic echocardiography, consisting of 1.5 h lecture and 1.5 h hands-on exercises on healthy volunteers. Teaching included the physical basics of ultrasound, the various cutting planes with focus on the apical 4-chamber view, the morphology and function of the left ventricle, normal and pathological left ventricular pump functions as well as the calculation of the ejection fraction. There was a maximum of four weeks between the introduction course and patient examinations.

All patient examinations were carried out in the recovery room in patients after general, trauma or heart surgery with a portable echocardiography device (VScan, GE, Solingen, Germany). Patient inclusion criteria were consent to echocardiography, ASA status I-IV, spontaneous breathing as well as the possible left lateral position and elevation of the left arm.

**Fig. 1:**
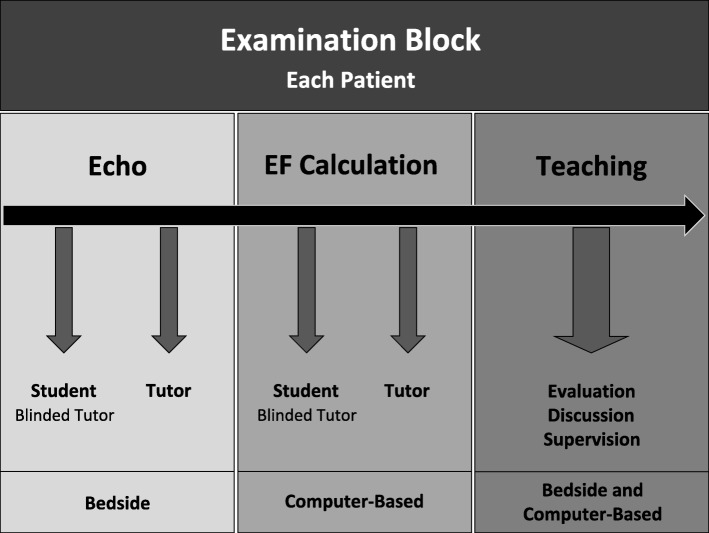
Examination Block

Each student performed a total of 10 *examination blocks*, one per patient (Fig. 1). Each *examination block* consisted of bedside echocardiography, where first the student (with blinded tutor) and then the tutor recorded three best possible loops of the apical four-chamber view to assess the left ventricular function. This was followed by the computer-based calculation of the ejection fraction (VScan Gateway Software, GE Healthcare, Solingen, Germany), where first the student (with blinded tutor) and then the tutor carried out the calculation. Finally, the bedside and computer-based teaching was held, which included the evaluation of imaging and calculation, discussion and supervision. Specifically, feedback was provided by evaluating position and angle of ultrasound transducer, determining the correct endocardial edge as well as defining end-diastole and end-systole. Using this formative approach, students were able to learn from their own mistakes. Both, the introduction course and all control examinations were carried out by the same tutor (TH).

### Measurements

The evaluation of the left ventricular function was performed as an *eye-balling method* and as a *calculated ejection fraction*. For the latter, both the diameter and the area of the left ventricle were measured, each end-diastolic and end-systolic. The volume was determined using the area-length formula (volume = 0.85 x Area^2^ / Diameter). Ejection fraction was calculated from the ratio of the stroke volume to end-diastolic volume. The *eye-balling method* and the ordinally scaled *calculated ejection fraction* used the categories *normal* (≥55%), *mildly abnormal* (45–55%), *moderately abnormal* (30–44%) or *severely abnormal* (< 30%) [20]. All measurements were carried out using a protocol shown in Table 1.

**Table 1:** Documentation parameters by students and tutor

Parameter	Item
Examination Quality	
Global Echocardiography Image Quality	Good, Medium, Bad
Complete Representation of Left Ventricle	Yes, No
Representation of the Cardiac Apex in the Top of the Ultrasound Sector	Yes, No
Segmental Analysis of Left Ventricular Walls ^a^	
Apical Septum	Normokinesia, Hypokinesia, Akinesia, Dyskinesia
Mid Inferoseptum	
Basal Inferoseptum	
Apical Lateral	
Mid Anterolateral	
Basal Anterolateral	
Ejection Fraction of Left Ventricle	
Eye-Balling ^a^	Normal, Mildly Abnormal, Moderately Abnormal, Severely Abnormal
Volumetry of Left Ventricle	
Length (end-diastolic)	[cm]
Area (end-diastolic)	[cm^2^]
Volume (end-diastolic) ^b^	[ml]
Length (end-systolic)	[cm]
Area (end-systolic)	[cm^2^]
Volume (end-systolic) ^b^	[ml]
Ejection Fraction of Left Ventricle	
Calculated ^c^	[%]

Documented parameters by the student and tutor with corresponding items for ordinal and dichotomous scaled variables (examination quality, segmental analysis of left ventricular walls, *eye-balling method* for determination of ejection fraction). ^a^ According to the recommendations for chamber quantification from the American Society of Echocardiography [20]. Length and area of left ventricle were measured. ^b^ Volume was calculated using the formula [volume = 0.85 x Area^2^ / Diameter]. ^c^ Ejection fraction was calculated using the formula [[end-diastolic volume - end-systolic volume] / end-diastolic volume]

### Statistical analysis

For ordinal scaled variables (global image quality, complete representation of left ventricle, representation of the cardiac apex in the top of the ultrasound sector, segmental analysis of left ventricular walls, ejection fraction of left ventricle with *eye-balling method* and *calculated*, ordinally scaled) the differences between student and tutor were expressed as percent matches given over the course of ten examination blocks. In order to estimate the bias of the measurements, additional positive or negative deviations were indicated. The increase in agreements between students and tutor in the course of the examination blocks was examined on the basis of the Spearman correlation. The two-sided significance level was defined at 5%. All data were tested for normal distribution (Shapiro-Wilk). We defined saturation of agreement when the number of examination blocks corresponded to at least 90% of linear fitted agreement.

Precision and bias for metric calculated diameter, area and ejection fraction of the left ventricle were assessed with Bland and Altman plots. The limit of agreement was defined as 1.96 times standard deviation. Additionally, we compared variances of student-tutor-differences for area, diameter and ejection-fraction between examination block 1 and 10 using the t-test statistics for two dependent samples, however, replacing means with variances. Statistical evaluation was performed with R software, version 3.2.3 (R Foundation for Statistical Computing).

## Results

25 students examined 250 patients, resulting in 500 transthoracic echocardiographies with 1.500 loops. No student and no patient were excluded from the study. Ten examination blocks of a single student were completed within one week.

During the course of training, the student-tutor-agreement for *global echocardiography image quality* significantly increased. All other items showed an improved agreement as well, without reaching significance. Using the *eye-balling method* to assess left ventricular function, students agreed with the tutor‘s findings both at the beginning (88%) but more at the end of training (95.7%). When the ejection fraction was *calculated*, the agreement between student and tutor was lower at the beginning (60%) than at the end of the course of training (91.3%). Both, *eye-balling method* and *calculation of ejection fraction* showed a trend towards a more precise determination, but they did not show any statistical significance. When the ejection fraction was *calculated*, the students tended to overestimate the pump function (bias + 11.7%). Using the linear fitted approach *global echocardiography image quality* reached saturation after at least 10, *calculated ejection fraction of left ventricle* after at least 14 examination blocks. Agreement, over- and underestimation of students with tutor in the assessment of the left ventricular function are presented in Table 2.

**Table 2:** Agreement, over- and underestimation of students with tutor in the assessment of the left ventricle

	Global Echocardiography Image Quality			Complete Representation of Left Ventricle			Representation of the Cardiac Apex in the Top of the Ultrasound Sector			Segmental Analysis of Left Ventricular Walls			Ejection Fraction of Left Ventricle					
													“Eye-Balling”			Calculated [ordinally scaled]		
Examination	Agreement, Over- and Underestimation of Students with Professional [%]																	
1	64.0	36.0	0.0	72.0	24.0	4.0	88.0	8.0	4.0	94.0	2.0	4.0	88.0	8.0	4.0	60.0	40.0	0.0
2	68.0	32.0	0.0	80.0	20.0	0.0	76.0	24.0	0.0	95.3	0.7	4.0	80.0	16.0	4.0	60.0	32.0	8.0
3	84.0	16.0	0.0	96.0	4.0	0.0	92.0	8.0	0.0	97.3	1.3	1.3	84.0	8.0	8.0	52.0	24.0	24.0
4	76.0	24.0	0.0	92.0	8.0	0.0	96.0	4.0	0.0	93.3	6.0	0.7	84.0	12.0	4.0	52.0	36.0	12.0
5	72.0	28.0	0.0	92.0	8.0	0.0	92.0	8.0	0.0	92.7	2.7	4.7	76.0	16.0	8.0	56.0	24.0	20.0
6	76.0	24.0	0.0	100.0	0.0	0.0	88.0	12.0	0.0	92.0	4.0	4.0	64.0	16.0	20.0	56.0	24.0	20.0
7	80.0	16.0	4.0	80.0	20.0	0.0	88.0	12.0	0.0	90.0	5.3	4.7	76.0	4.0	20.0	60.0	20.0	20.0
8	76.0	24.0	0.0	96.0	4.0	0.0	100.0	0.0	0.0	99.3	0.7	0.0	80.0	16.0	4.0	84.0	8.0	8.0
9	95.8	4.2	0.0	95.8	4.2	0.0	95.8	4.2	0.0	93.7	0.7	5.6	91.7	0.0	8.3	70.8	25.0	4.2
10	100.0	0.0	0.0	100.0	0.0	0.0	100.0	0.0	0.0	96.4	0.0	3.6	95.7	0.0	4.3	91.3	4.3	4.3
Bias	+ 20.0%			+ 8.8%			+ 7.6%			−0.009%			+ 1.1%			+ 11.7%		
Spearman r	0.75			0.61			0.64			−0.006			0.16			0.62		
*p* value	0.002			0.067			0.054			1.0			0.657			0.06		

Percentage agreement, over- and underestimation of students with the tutor in the presentation of the left heart in echocardiography and the assessment of left ventricular function in the course of ten examination blocks. Overestimation or positive bias means poorer global image quality, more often insufficient representation of left ventricle, more frequent lack representation of the cardiac apex in the top of the ultrasound sector or a worse judged wall movement by the student compared to the tutor, underestimation or negative bias the opposite. Spearman r and p-value were calculated from the correlation between the examination number and the agreement between student and tutor

**Fig. 2:**
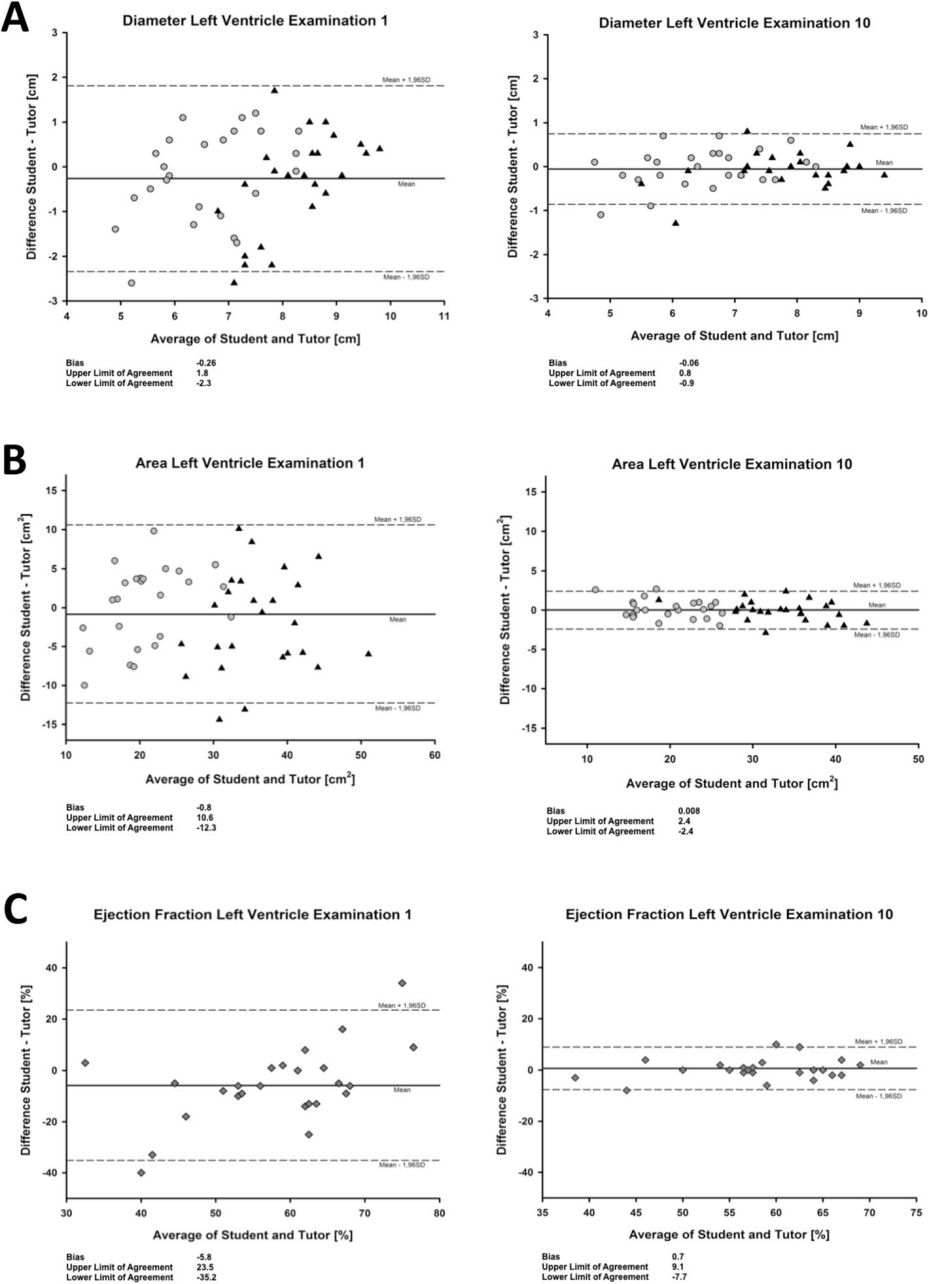
Bland and Altman plots of the respective first and tenth examination blocks for measurements of left ventricular diameter (**a**) and area (**b**) (end-diastolic marked as triangles, end-systolic marked as circles), both needed for calculation of ejection fraction (**c**). The plots show the measured differences between student and tutor (y-axis) depending on the respective average (x-axis). The precision increases for both the determination of the diameter and the area between the first and tenth examination block. The calculation of the ejection fraction also follows this trend. The solid lines represent the bias (mean), the dashed lines the limits of agreement (mean ± 1.96 SD). For comparability, the scales of the y-axes are identical for the first and tenth examinations

The variation of student-tutor-differences for calculation of area, diameter and ejection fraction, respectively, was significantly lower in examination block 10 than in examination block 1 (each of the two-sided *p*-values < 0.001). Bland and Altman plots in Fig. 2 show the improved precision and the respective bias with limits of agreement of the students in the tenth compared to the first examination block for left ventricular diameters, areas, and calculated ejection fractions.

## Discussion

A structured supervised course of training in focused echocardiography for last year medical students results in an improved global image quality and a more accurate assessment of left ventricular function. Latter applies to both the *eye-balling method* and in particular to the accurate *calculation of the left ventricular ejection function*. Although significance levels were not reached in all items, the clinical and educational effects are visible and relevant.

We obtained these results from 500 echocardiography studies. High agreement rates between tutor and students already at the beginning of the course using the *eye-balling method* suggests that even a theoretical lesson allows a rough estimation of the left ventricular function. This is different for the actual calculation. At the beginning, the student often fails to fully visualize the left ventricle and edge the endocard in the echocardiographic sector leading to underestimation of the ventricular diameter and area. This becomes clear from the negative bias in both the determination of the diameter and the area in the first examination block (Fig. 2). As a result, the student fails to accurately calculate the ejection fraction. However, this measurement error was much less in the tenth examination block, leading to a more precise calculated ejection fraction.

We used a linear approach with at least 90% of agreements to detect saturation. We are aware of the high variability especially in the last examination blocks. However, more precise methods like three-dimensional echocardiography or three-dimensional cardiac magnetic resonance imaging show frequently worse agreements with two-dimensional transthoracic echocardiography compared to our student-tutor-differences [21]. Malm and colleagues demonstrated both, volume and ejection underestimation by transthoracic echocardiography compared to the gold standard cardiac magnetic resonance imaging. They reported a bias of − 56 ml (± 48 ml for the limits of agreement) for calculation of end-diastolic volume, − 16 ml (± 32) for end-systolic volume and − 6% (±14) for calculation oft the ejection fraction. Moreover, mean inter-observer variability for calculation of ejection fraction was 13.9%, mean intra-observer variability 5.4% [22]. Regarding our differences between students and tutor in calculation of ejection fraction especially in the tenth examination block, we report limits of agreement between – 7.7 and 9.1% comparable to the previous mentioned findings. Again, this residual error might be attributed of course to worse skills of students compared to the tutor but also to the inaccuracy of transthoracic echocardiography itself as well as inter- and intra-observer variability.

Numerous studies have already shown that structured echocardiography training for students and residents improves theoretical knowledge and practical skills [6, 23]. In particular, clear learning success by a structured training program could be shown for rough assessment of pump function [9, 12, 14]. Hope et al. showed that a visual approach using template matching led to a sufficient categorical assessment of left ventricular function with minimal training in students [10]. Nevertheless, the didactics were very different between all studies.

A theoretical introduction is indispensable for the basic understanding of anatomy and physiology. However, the practical training on patients seems to determine the learning success. We demonstrated that a structured course of training significantly improves the precision of metric determination of left ventricular diameter and area, both needed for exact calculation of left ventricular ejection fraction. This effect is noticeable already after ten supervised echocardiographic examination blocks. Our data suggest that on average at least 14 examination blocks are necessary to achieve 90% agreement of correct *calculation of left ventricular ejection fraction*. Nevertheless, it must be mentioned that repetitive and long-term training is required to keep up to the same level of performance and maintain high quality in transthoracic echocardiography.

We consciously selected students with neither prior theoretical knowledge nor practical experience in transthoracic echocardiography. And even for this study population a learning success could be shown. Probably the correct measurement of left ventricular diameter and area is one of the most demanding methods in transthoracic echocardiography especially at the beginning. However, this method allows measuring the learning success quantitatively by comparing diameter and area between student and tutor.

Our cardiac ultrasound course of training followed a formative approach. In contrast to a summative procedure, teaching was combined with direct assessment of the knowledge and skills in each individual examination block. As a result, the students received feedback not just at the end of the course of training, so that errors could be corrected directly and assistance could be implemented immediately. Thus, the greatest possible learning success could be ensured.

Perhaps conventional ultrasound imaging devices would have led to a better image quality and thus to a simpler understanding of anatomy and physiology. The use of pocket-sized ultrasound devices allows more flexible and mobile use. Furthermore, numerous studies have demonstrated that hand-held devices can be easily used in clinical routine and especially in student education [11–13, 24–26].

### Limitations

Our study has several limitations. First, determination of the left ventricular ejection fraction may be an inappropriate outcome parameter. For example, both underestimated end-diastolic and end-systolic volumes by students might result in a correct ejection fraction. Nevertheless, the improvement of precision is especially detectable for the ventricular diameter and the area. Thus, correct determination of ejection fraction is not a coincidence, but the result of a more precise measurement of area and diameter during the course of training. Second, the ejection fraction was measured only mono- but not biplane. However, this would have necessitated the inclusion of another transthoracic plane and possibly overwhelmed the students in this setting. Third, the training focused only on the determination of the left ventricular ejection fraction. The detection of heart valve defects or other pathologies has not been considered, but could be closely related. Fourth, the majority of patients had no major cardiac pre-existing conditions with mostly normal pump function. Nevertheless, the principle of measurements should not be affected. It also has to be considered critically that the students only carried out ten examination blocks. An even higher number could possibly have shown an even stronger effect with a lower variability. In case number planning, we have based our own clinical experience in education as well as previously published work in this field [11, 12, 27]. And finally, only one tutor supervised all students. Thus, he might have been aware of the design and might have expected outcomes and results. Moreover, we are aware that a period of up to four weeks between theoretical introduction and examination blocks might have an individual impact on learning success. Due to the small number of students we were not able to adjust the learning success to this confounder.

We did not evaluate any basic knowledge prior to the theoretical three-hour lesson, nor did we retrospectively review the theoretical learning success. Moreover, we did not know if and to what extent the theoretical training improved the practical implementation. Maybe this normalization would have affected the results. However, we hypothesized that the theoretical knowledge and practical skills in transthoracic echocardiography were initially marginal in last year medical students, so the initial conditions were similar. Beside that, long-term retention was not assessed and thus the durability of the improvement in precise measurement of left ventricular ejection fraction cannot be assessed in this study. Therefore, the improvement shown in our present data may be lost over time [28].

Future research is needed to determine if this learning concept can be integrated with other transthoracic echocardiography means into the curriculum of medical schools.

## Conclusions

A structured theoretical and practical transthoracic echocardiography course of training for last year medical students provides a measurable learning experience for the assessment and calculation of left ventricular pump function. Incorporating training of transthoracic echocardiography in medical student education may be one step further towards a more widespread use of ultrasound for many specialties.

## Data Availability

The datasets used and/or analysed during the current study are available from the corresponding author on reasonable request.

## Abbreviations

EF: Ejection fraction
GLS: Global longitudinal strain
VTI: Velocity time integral
